# Academic Leagues and the Formation of Physicians in Brazil

**DOI:** 10.1590/1516-3180.2026.1444.02032026

**Published:** 2026-08-21

**Authors:** Alberto Jorge Monteiro Dela Vega, Paulo Manuel Pêgo-Fernandes

**Affiliations:** I MD, PhD. Attending Physician, Thoracic Surgery Service, Instituto do Cancer do Estado de São Paulo, Hospital das Clinicas HCFMUSP, Faculdade de Medicina, Universidade de Sao Paulo, Brazil.; II MD, PhD. Full Professor, Thoracic Surgery Program, Instituto do Coração, Hospital das Clínicas HCFMUSP, Faculdade de Medicina, Universidade de São Paulo, São Paulo (SP), Brazil; Director, Scientific Department, Associação Paulista de Medicina (APM), São Paulo (SP), Brazil.; Scientific Department, Associação Paulista de Medicina (APM), São Paulo, SP, Brazil

Medical education has traditionally emphasized the acquisition of theoretical knowledge and procedural competence as the central attributes of physician training. However, contemporary practice increasingly demands abilities that extend beyond technical expertise, including communication, teamwork, leadership, ethical responsibility, and engagement with healthcare systems and research. This gap between formal curricula and the broader professional role expected of physicians has become a persistent challenge in medical education worldwide.

The CanMEDS competency framework proposed by the Royal College of Physicians and Surgeons of Canada formalized this expanded understanding of the physician’s role, defining the doctor not only as a medical expert but also as a communicator, collaborator, leader, scholar, health advocate, and professional. Competency-based medical education therefore requires learning environments in which these roles can be experienced in practice rather than only taught in theory.^1^ ^,^ ^2^

Within Brazilian medical education, academic leagues have emerged as a distinctive response to this need. These student-led organizations complement formal curricula by integrating teaching activities, research participation, and supervised clinical exposure. Their widespread presence across medical schools reflects both student demand and institutional recognition of their educational relevance. In this context, leagues function not merely as extracurricular groups, but as environments in which students transition from passive recipients of information to active participants in professional formation.

 A comprehensive survey revealed that in the setting of 126 Medical Schools, only four didn’t have information about AL. All public medical schools and 96.2% of private institutions host academic leagues, with 74.4% maintaining specialized leagues in high-demand areas such as oncology. This widespread adoption reflects genuine student demand and institutional recognition of their educational value. The digital presence of these organizations is substantial, with the majority maintaining active social media profiles and discussion forums, demonstrating their adaptation to contemporary educational technologies.^3^

Evidence supports this educational role. Studies evaluating pain education in Brazil demonstrated that many institutions lack structured curricular coverage of important clinical topics, whereas leagues provide organized teaching activities, supervised patient care, and research participation. International literature similarly shows that structured academic extracurricular activities are associated with leadership development, increased self-confidence, improved academic satisfaction, and greater postgraduate engagement. These findings suggest that such initiatives can partially compensate for curricular limitations while fostering early professional identity formation.^4^

The experience of the Cardiothoracic Surgery Academic League at the University of São Paulo Medical School illustrates this model in practice. Operating continuously for more than two decades, the league integrates lectures, research activities, and clinical observation. Its annual introductory course, organized primarily by students and attracting approximately 60 applicants each year, functions both as an educational event and as the selection process for new members. The preparation of this course requires coordination, communication, and responsibility among students, transforming the organizational process itself into a learning experience. Beyond clinical exposure, participation has been associated with scientific production and influence on specialty choice, demonstrating its impact on professional development.

In a article published in 2010 the league showed that they had 47 scientific publications with league members as authors or co-authors. Beyond publications, the league has successfully integrated teaching, research, and community engagement through structured activities including theoretical lectures, scientific meetings, and practical observation in the operating room and post-operative recovery units. Student satisfaction with this model was exceptionally high. Among current members, 78.5% responded to evaluation surveys, with 98% recommending the league to colleagues. Notably, 49% of league participants engaged in scientific research projects directly linked to the league, demonstrating its capacity to foster research engagement.^5^

Early and sustained contact with a specialty increases the likelihood that students will pursue it during postgraduate training. This effect is not merely the result of increased exposure, but of meaningful engagement: students encounter mentors, observe professional identities in practice, and begin to envision their own place within the field. In this sense, academic leagues function as more than educational initiatives. They contribute to workforce formation by attracting motivated students to areas that require long training and early commitment, such as surgical specialties. By facilitating mentorship and guided participation, leagues help align professional development with healthcare needs, reinforcing both individual vocation and social responsibility.

Academic leagues therefore represent more than complementary activities; they constitute practical environments of competency-based education. By promoting mentorship, teamwork, research engagement, and responsibility for collective tasks, they anticipate principles later formalized in modern educational frameworks. Rather than being viewed as informal student initiatives, such structures should be recognized as valuable components of medical training. Their experience suggests that meaningful professional formation may depend not only on curricular reform, but also on preserving learning spaces in which students actively participate in the practice, organization, and social role of medicine.
